# 1,2-Dimyristoyl-*sn-glycero*-3-phosphocholine promotes the adhesion of nanoparticles to bio-membranes and transport in rat brain†

**DOI:** 10.1039/d1ra01737c

**Published:** 2021-11-03

**Authors:** Dong Han, Baolin Zhang, Jianghui Dong, Boning Yang, Yuntao Peng, Junfeng Wang, Liping Wang

**Affiliations:** College of Materials Science and Engineering, Key Laboratory of Nonferrous and Materials Processing Technology, Ministry of Education, Guangxi Key Laboratory of Optical and Electronic Materials and Devices, Guilin University of Technology Guilin 541004 Guangxi China zhangbaolin@glut.edu.cn; Guangxi Engineering Research Center of Digital Medicine and Clinical Translation, College of Biotechnology, Affiliated Hospital of Guilin Medical University, Guilin Medical University Guilin Guangxi 541004 China; Guangxi Collaborative Innovation Center for Biomedicine, Department of Human Anatomy, Guangxi Medical University Nanning 530021 Guangxi China

## Abstract

1,2-Dimyristoyl-*sn-glycero*-3-phosphocholine (DMPC) coated on the surface of superparamagnetic iron oxide nanoparticles (SPIONs) has advantages in neurotherapy and drug delivery. In this study, the surface of polyvinylpyrrolidone (PVP)-SPIONs was modified with DMPC, then PVP-SPIONs and DMPC/PVP-SPIONs were co-incubated with rat adrenal pheochromocytoma (PC-12) cells to observe the effect of DMPC on the distribution of SPIONs in cells, and further PVP-SPIONs and DMPC/PVP-SPIONs were implanted into the substantia nigra of Sprague-Dawley (SD) rats by stereotaxic injection, and the brain tissues were removed at both twenty-four hours and seven days after injection. The distribution and transport of nanoparticles in the substantia nigra *in vivo* were explored in these different time periods. The results show that DMPC/PVP-SPIONs were effectively distributed on the membranes of axons, as well as dendritic and myelin sheaths. The attachment of nanoparticles to bio-membranes in the brain could result from similar phospholipid structures of DMPC and the membranes. In addition, DMPC/PVP-SPIONs were transported in the brain faster than those without DMPC. *In vitro* experiments found that DMPC/PVP-SPIONs enter cells more easily. These characteristics of iron oxide nanoparticles that are modified by phospholipids lead to potential applications in drug delivery or activating neuron membrane channels.

## Abstract 2

1,2-Dimyristoyl-*sn-glycero*-3-phosphocholine (DMPC) coated on the surface of superparamagnetic iron oxide nanoparticles (SPIONs) has advantages in neurotherapy and drug delivery.

## Graphical abstract

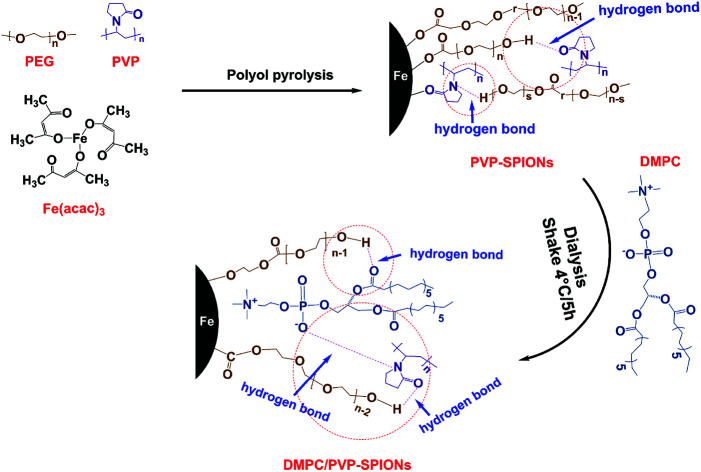

## Introduction

Parkinson's disease (PD) is a chronic, neurodegenerative disease, usually caused by the degeneration or death of dopaminergic neurons in the substantia nigra of the brain, causing abnormal motor function and leading to striatal dopamine (DA) deficiency. Levodopa can treat PD, but it might only provide short-term benefits and may be limited by its side effects. Long-term drug treatments can lead to neurological complications.^1,2^ Deep brain stimulation, combined with implantable magnetic nanoparticles, can shorten the effective time and reduce the required dose of levodopa. Bio-conjugated superparamagnetic iron oxide nanoparticles (SPIONs) can be used in neurology, similar to activating neuron membrane channels, for targeted treatment of brain tumours and magnetic resonance imaging (MRI) contrast agents, owing to its good biocompatibility and special magnetic properties.^3–5^ Under the control of external magnetic fields, nerve behaviour in the brain can be regulated by the effective and targeted distribution of magnetic nanoparticles.^6^ This method can activate heat-sensitive capsaicin receptor TRPV1 by the magneto-calorific effects under an alternating current magnetic field (ACMF), which triggers reversible neuronal activation and protects dopamine neurons by the remote control of neuron excitation.^7–9^ This strategy promotes the treatment of neurological disorders with dopamine deficiency. However, the distribution and adsorption of nanoparticles on the bio-membrane *in vivo* are still unclear.

Polymer materials coated on SPIONs can facilitate colloid stability and provide functional groups, such as terminal amines or carboxyl groups, for the design of multifunctional SPIONs for a range of applications.^1,2,10^ Suitable SPIONs modified with a functional molecule help to establish an effective connection between SPIONs and biological systems.^11–13^ For example, Mannix *et al.*^14^ reported that superparamagnetic beads covered by multivalent integrin ligands linked to a single integrin receptor on the membrane surface and agglomerated into clusters were able to activate signal transduction in the presence of a magnetic field. There is also a report that tail-vein-injected Tween-80 surface-modified monodispersed SPIONs moved across the intact blood–brain barrier (BBB) of rats under a magnetic field.^15^

Phospholipids, integral components of cell membranes, are composed of polar head groups and nonpolar hydrocarbon tails.^16^ Therefore, the use of phospholipids as a surface modifier can facilitate the synthesis of biocompatible nanomaterials and cause the nanoparticles to target the bio-membrane. 1,2-Dimyristoyl-*sn-glycero*-3-phosphocholine (DMPC) is a long double carbon chain.^17^ It can be used as a functional molecule for targeting cell membranes due to its good biocompatibility, biodegradability, the similarity with the cell membrane structures, and low immunological responses.^18^ Poly(vinylpyrrolidone) (PVP) is an amphiphilic polymer which is able to inhibit protein adsorption onto surfaces. Similar to polyethylene glycol (PEG), PVP can inhibit the nonspecific interaction between nanoparticles and plasma proteins, reduce the clearance by endothelial reticular system and prolong the blood circulation time.^19^

In this study, hydrosoluble PVP-SPIONs were prepared by polyol pyrolysis using PEG as a solvent and reagent,^20–22^ PVP as an additive, and iron(iii) acetylacetonate (Fe(acac)_3_) as an iron source. A high temperature led to rapid nucleation and growth of the newly formed particles, resulting in uniform small-sized SPIONs.^1^ The surface of PVP-SPIONs was modified with DMPC, and then PVP-SPIONs and DMPC/PVP-SPIONs were co-incubated with rat adrenal pheochromocytoma (PC-12) cells with neuronal characteristics to observe the effect of DMPC on the distribution of SPIONs in cells. Furthermore, SPIONs were implanted into the substantia nigra of SD rats by stereotaxic injection, and brain tissues were removed at both twenty-four hours (24 h) and seven days (7 d) after injection. By comparing the results with extracorporeal cell uptake, the distribution and transportation process of PVP-SPIONs and DMPC/PVP-SPIONs were investigated.

## Materials and methods

### Synthesis of DMPC/PVP-SPIONs

**Fig. 1:**
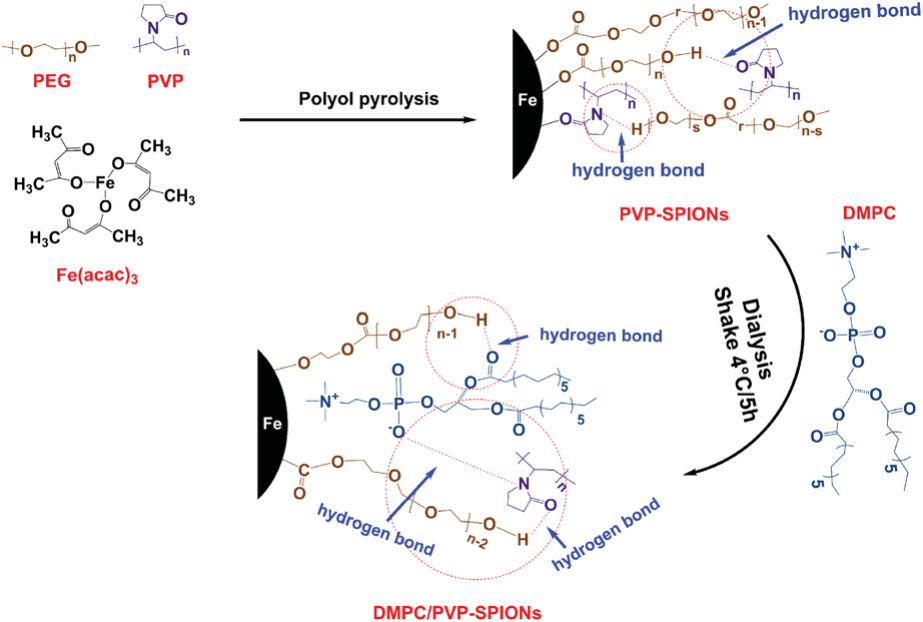
Schematic diagram of the synthesis of PVP-SPIONs and DMPC/PVP-SPIONs.

To prepare stably dispersed DMPC/PVP-SPIONs, PVP-SPIONs were synthesized by polyol pyrolysis, the detailed synthesis of the PVP-SPIONs can be found in the ESI.† Then DMPC was modified on PVP-SPIONs by hydrogen bonding mediated physical adsorption (Fig. 1). 15 mg of DMPC was added to 20 mL of PVP-SPIONs (about 2 mg mL^−1^) in aqueous dispersion. After which, DMPC was placed in a shaking table set at 100 rpm for five hours, in 4 °C. After five hours, the DMPC/PVP-SPIONs were collected by using LS columns (Miltenyi Biotec, Germany) and were thoroughly washed with a 0.01 M PBS buffer solution, this process was repeated three times to remove residue and free DMPC.

### Cytotoxicity assay *in vitro*

PC-12 cells were bought from the Stem Cell Bank of the Chinese Academy of Sciences (Shanghai, China). The PC-12 cell line was obtained from transplantable male rat adrenal pheochromocytoma. PC-12 cells can form dopamine and show choline acetyltransferase and tyrosine hydroxylase. Thus, they are widely used in the study of nerve cell function.^23–25^ The steps in cell culture after resuscitation are shown in the ESI;† Fig. S1† shows the state of the cells after resuscitation. A hemocytometer was used to count the number of cells that returned to normal. The cytotoxicity assay of SPIONs *in vitro* was assessed by using CCK-8 activity detection.

After cell counting, the cells were incubated on 96-well microtiter plates with 7 × 10^3^ cells per well and added to 100 μL of RPMI 1640 complete medium per well for 24 h. Then the RPMI 1640 medium was sucked out, and 100 μL of PVP-SPIONs, DMPC/PVP-SPIONs were added to each well at several concentrations (0, 5, 10, 25, 50, 100, 200 μg mL^−1^, dispersed in 0.01 M PBS buffer with pH = 7.2). Nanoparticles of each concentration are added to 6 wells to increase the reliability of the test results when counting the optical density (OD) value. Following incubation for 24 h, 48 h, and 72 h, each well was first filled with 0.01 M PBS buffer of 100 μL for blow cleaning. Because most cells are suspended at this time, they need to be centrifuged (1500 rpm for 10 min) at the bottom by using a desk-top speed centrifuge (Optima MAX-P, USA). Then the 0.01 M PBS buffer was removed, 100 μL CCK-8 was put into each well and set in the carbon dioxide incubator, the cytotoxicity of SPIONs was examined by ELISA Microplate Reader 3 h later.

### Cellular uptake experiments with SPIONs

Cellular uptake experiments were carried out in 6-well cell culture plates with a density of 5 × 10^5^ per well, and cells were incubated in RPMI 1640 complete medium for about 24 h. The RPMI 1640 medium, having 200 μg mL^−1^ PVP-SPIONs, DMPC/PVP-SPIONs (dispersed in 0.01 M PBS buffer with pH = 7.2), was added to the 6-well plate and cultured for 12 h. Then the cells were centrifugally obtained in 15 mL plastic centrifuge tubes (1000 rpm for 10 min), and the excess nanoparticles were rinsed by 0.01 M PBS buffer in the intermediate process. Finally, 3% glutaraldehyde was used to immobilize the cells, and for storage at 4 °C.

### Animal experiment

All the experiments in this study were performed according to the guidelines of the Experimentation Ethics Committee of the Guilin Medical University, China (approval no. 2020-0015). Adult male Sprague-Dawley (SD) rats (250–270 g, SPF grade) were purchased from Hunan SJA Laboratory Animal Co, Ltd (Changsha, China). The SD rats were anesthetized with an intraperitoneal injection of 2% pentobarbital (45 mg kg^−1^), and they were considered to be prepared for the experiment when receiving intense pain stimulation in the hindfoot without an obvious muscle contraction reaction. All SD rats were fed with standard pellet feed and water under relative humidity (40% to 50%) and temperature (22 ± 2 °C).

18 rats were randomly divided into three groups (*n* = 6 in each group): control group (CON): 0.9% saline (10 μL) was injected into the left side of the substantia nigra of SD rats; PVP-SPIONs group: PVP-SPIONs solution (1 mg mL^−1^, 10 μL, dispersed in 0.01 M PBS buffer with pH = 7.2) was injected into the left side of the substantia nigra of SD rats; DMPC/PVP-SPIONs group: DMPC/PVP-SPIONs solution (1 mg mL^−1^, 10 μL, dispersed in 0.01 M PBS buffer with pH = 7.2) was injected into the left side of the substantia nigra of SD rats.

The head of the rat was fixed on a stereotaxic device, and the microsyringe and rat brain were placed on the same horizontal line. According to the location of the left side of the substantia nigra (coordinate: AP, −5.2; ML, 2.3; DV, 7.6 mm) in the SD rat, a hole was drilled into the skull with a diameter of 2 mm at the “target point”. The polyethylene tubing and 25 μL microsyringe was filled with the appropriate amount of PVP-SPIONs, DMPC/PVP-SPIONs (dispersed in 0.01 M PBS buffer with pH = 7.2). A micropump (KDS100, USA) was used as the pressure injection equipment. The substantia nigra was injected with SPIONs at a speed of 1 μL min^−1^. After injection, the original state was maintained for 10 min, and the rats were moved to a mild environment after the wound was sutured.

At 24 h and 7 d after injection, the brains of SD rats were taken and stored in paraformaldehyde (PFA, 4%) after cardiac perfusion with 500 mL 0.9% saline and fixation with 500 mL PFA (4%).

### TEM observation of SPIONs distribution

Immobilized PC-12 cells and substantia nigra tissue samples (∼1 mm^3^) were embedded in epoxy resin after a series of treatments (ESI†). The samples were sliced into 60–70 nm flakes by ultramicrotome (Power Tome-XL, USA) and loaded on a common copper network (200 mesh) for double staining (ESI†). The biodistribution of SPIONs in the substantia nigra and intracellular space was observed by two types of TEM instruments. One was the H-7650 (Japan, voltage: 80 kV), which is mainly used to observe the biodistribution of SPIONs in organisms. The other was the JEM-2100F (Japan, voltage: 200 kV) that is mainly used to observe SPIONs morphology and lattice fringes, and it is also equipped with energy-dispersive X-ray spectroscopy (EDS) for the quantitative analysis of elements.

### Statistical analysis

Data are presented as the mean ± SD. In each experiment, *n* represents the number of independent experiments or the number of animals per group. One-way ANOVA was used to compare multiple-group values (*i.e.*, viability tests of cells). Statistical significance of iron contents of regions was assessed by one-way ANOVA and *post hoc* comparison using Fisher's least significant difference test, *p < 0.05; **p < 0.01; ***p < 0.001.

## Results and discussion

### Characterization of the SPIONs

**Fig. 2:**
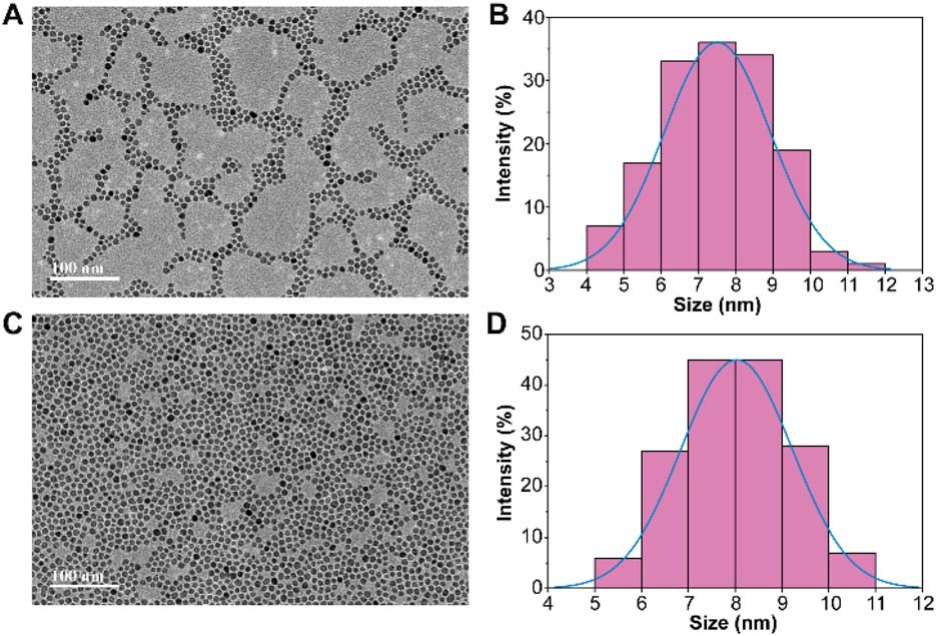
The morphology and size distribution of PVP-SPIONs (A and B), and DMPC/PVP-SPIONs (C and D).

Fig. 2 shows the morphology and size of the PVP-SPIONs and DMPC/PVP-SPIONs with good dispersion. The average particle sizes of the PVP-SPIONs and DMPC/PVP-SPIONs were 7.5 ± 1.4 nm and 8.0 ± 1.2 nm, respectively, which were measured and calculated using ImageJ software. The hydrodynamic diameters of the PVP-SPIONs and DMPC/PVP-SPIONs were 23.8 nm and 110.4 nm, respectively (Table 1). The zeta potential indicates that the DMPC/PVP-SPIONs had a weak negative electrical charge (−4.2 mV) in 0.01 M PBS buffer when DMPC was modified on the PVP-SPIONs whose zeta potential was 0 mV.

**Table 1:** Hydrodynamic diameter and the zeta potential of PVP-SPIONs and DMPC/PVP-SPIONs

	PVP-SPIONs	DMPC/PVP-SPIONs
Hydrodynamic diameter (nm)	23.8	110.4
Zeta potential (mV)	0	−4.2

**Fig. 3:**
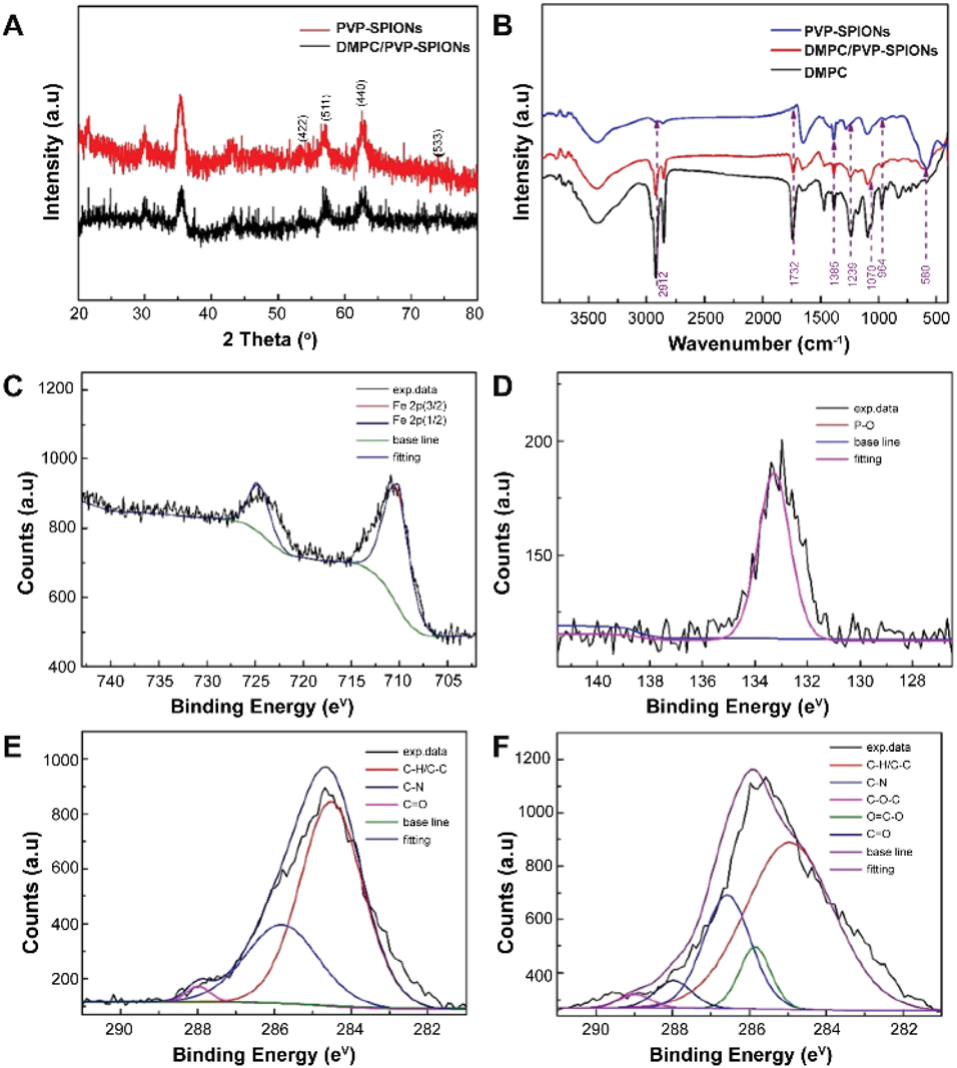
XRD spectra (A), FT-IR spectra (B), XPS spectra: Fe 2p of DMPC/PVP-SPIONs (C), P 2p of DMPC/PVP-SPIONs (D), C 1s of PVP-SPIONs (E), and C 1s of DMPC/PVP-SPIONs (F).

The XRD spectra of the nanoparticles matched the crystal planes of magnetite (Fe_3_O_4_) crystal (Fig. 3A). The result shows that the diffraction peaks that appear at 29.9°, 35.4°, 43.1°, 53.4°, 56.9°, 62.6°, 74.0° are basically the same as those of the standard cards of Fe_3_O_4_ PDF (JCPDS 01-085-1436), which corresponds to (220), (311), (400), (422), (511), (440), and (533) crystal planes show the main crystal phase of the SPIONs.

The FT-IR spectroscopic analysis (Fig. 3B) shows that both DMPC and DMPC/PVP-SPIONs have strong absorption peaks at 1239 cm^−1^ and 1070 cm^−1^, which can be attributed to the stretching vibration of P–O.^26^Fig. 3C shows that the main component of nanoparticles is iron. The XPS 133.3 eV peak of P 2p^27,28^ proves that DMPC had bonded on the surface of PVP-SPIONs (Fig. 3D). Fig. 3E and F show that XPS of those functional groups also confirms that the nanoparticles have been modified with DMPC (ESI†). As it is shown in Fig. 1 that the C=O and P–O^−^ on the DMPC are combined to the polymer by hydrogen bonding, so that the DMPC is firmly adsorbed on the surface of the PVP-SPIONs.

**Fig. 4:**
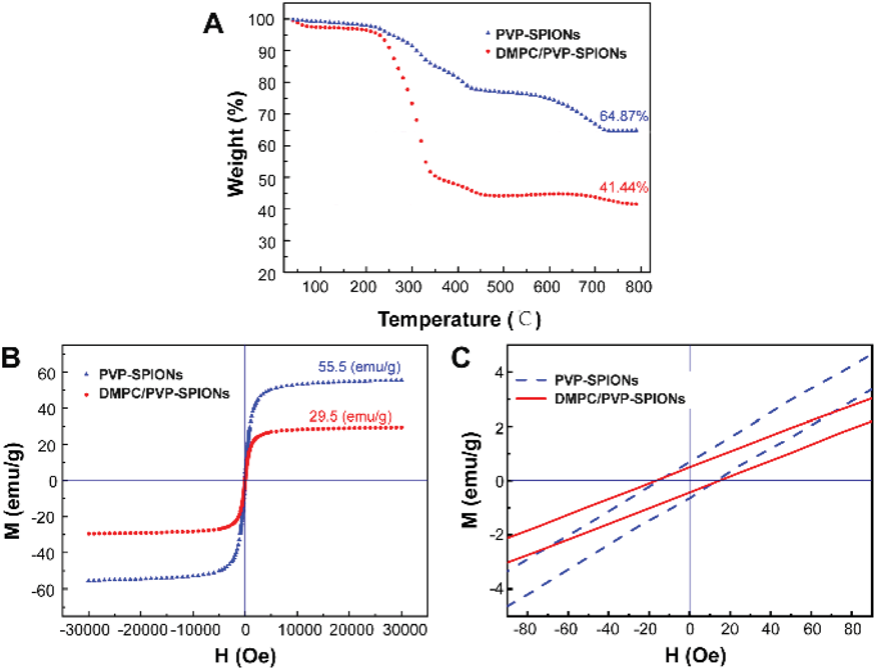
TGA curves of PVP-SPIONs and DMPC/PVP-SPIONs (A), the hysteresis *M*–*H* curves of PVP-SPIONs and DMPC/PVP-SPIONs in 300 K (B and C).

The TGA curves of the PVP-SPIONs and DMPC/PVP-SPIONs show that the weight loss of the PVP-SPIONs and DMPC/PVP-SPIONs were 35.13% and 58.56%, respectively (Fig. 4A). The amount of DMPC modified on PVP-SPIONs was calculated to be 36.12%. The hysteresis *M*–*H* curves showed the saturated magnetizations of the PVP-SPIONs and DMPC/PVP-SPIONs were 55.5 emu g^−1^ and 29.5 emu g^−1^, respectively (Fig. 4B). Both nanoparticles exhibited low coercivity (<20 Oe) at 300 K (Fig. 4C); these nanoparticles are generally considered to be superparamagnetic as long as the coercivity is less than 30 Oe.^29^ The non-zero coercivity may be caused by the agglomeration of SPIONs during freeze-drying.

### *In vitro* cytotoxicity of SPIONs

**Fig. 5:**
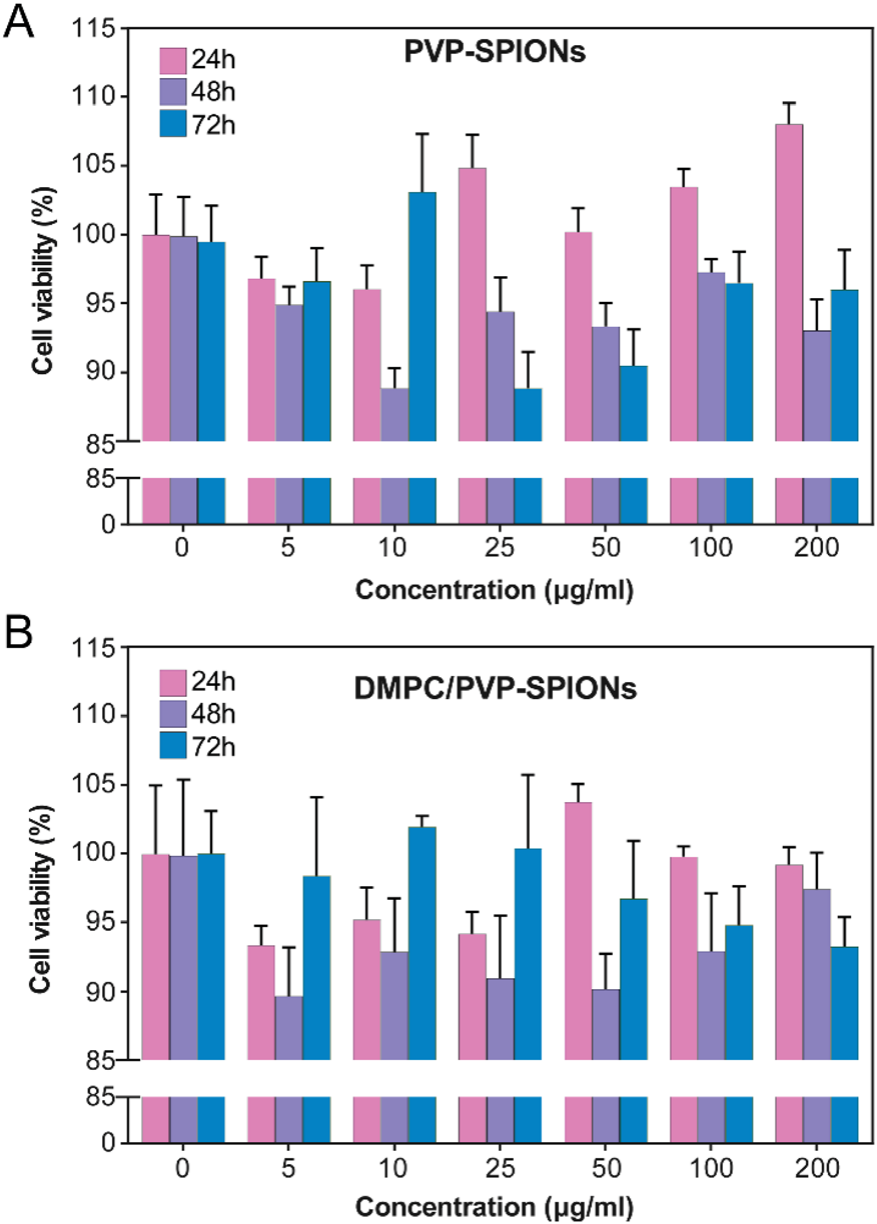
Viability tests of PC-12 cells after incubation with 0–200 μg mL^−1^ PVP-SPIONs (A) and DMPC/PVP-SPIONs (B) for 24–72 h.

*In vitro* cytotoxicity of PVP-SPIONs and DMPC/PVP-SPIONs at different concentrations (5–200 μg mg^−1^) and times (24–72 h) was measured by using CCK-8. Compared with the CON (0 μg mL^−1^), PVP-SPIONs and DMPC/PVP-SPIONs had hypotoxicity, and the cell viabilities were more than 90% (Fig. 5). Individual concentrations showed high cell viability due to the PVP/PEG-modified nanoparticles, improving biocompatibility with an increase in the amount of PVP/PEG with significantly promoted cell growth and proliferation.^30^

### Subcellular distribution of the SPIONs

**Fig. 6:**
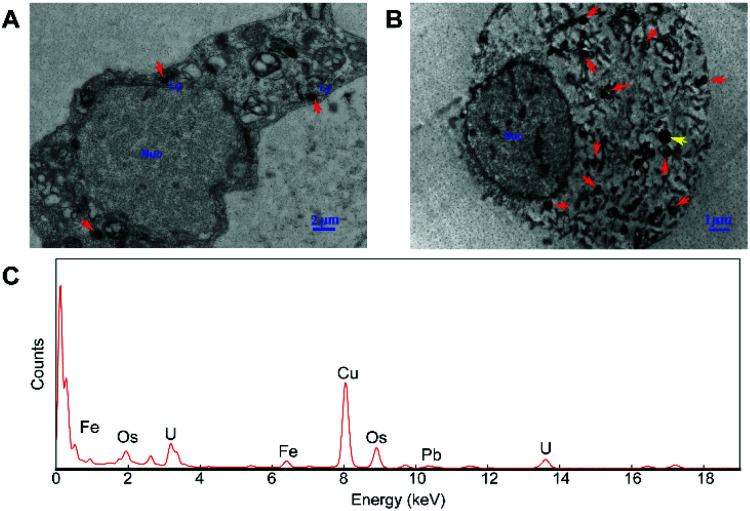
The distribution of PVP-SPIONs (A) and DMPC/PVP-SPIONs (B) in PC-12 cells, and EDS elemental analysis (C).

In order to explore the subcellular distribution of the DMPC modified PVP-SPIONs in PC-12 cells and in the substantia nigra in rat brains, ultrathin sections were made of collected cells and left substantia nigra tissue samples to observe the biological distribution of SPIONs by TEM. Fig. 6A and B show the subcellular distribution of PVP-SPIONs and DMPC/PVP-SPIONs after incubation with PC-12 cells for 12 h. It was found that the amount of DMPC/PVP-SPIONs entering the PC-12 cells was significantly larger than that of PVP-SPIONs (the nanoparticles are labeled with red arrows). DMPC/PVP-SPIONs were mainly distributed in the cell cavity, lysosome, and vesicle. The dense black cluster marked by yellow arrows in Fig. 6B was analyzed by EDS. Fig. 6C shows a strong peak of Fe Kα, indicating that the black particles contain SPIONs. The results of the *in vitro* experiments show that DMPC distinctly enhances the ability of nanoparticles to enter cells because of the similar phospholipid structures of DMPC and the cell membrane.

**Fig. 7:**
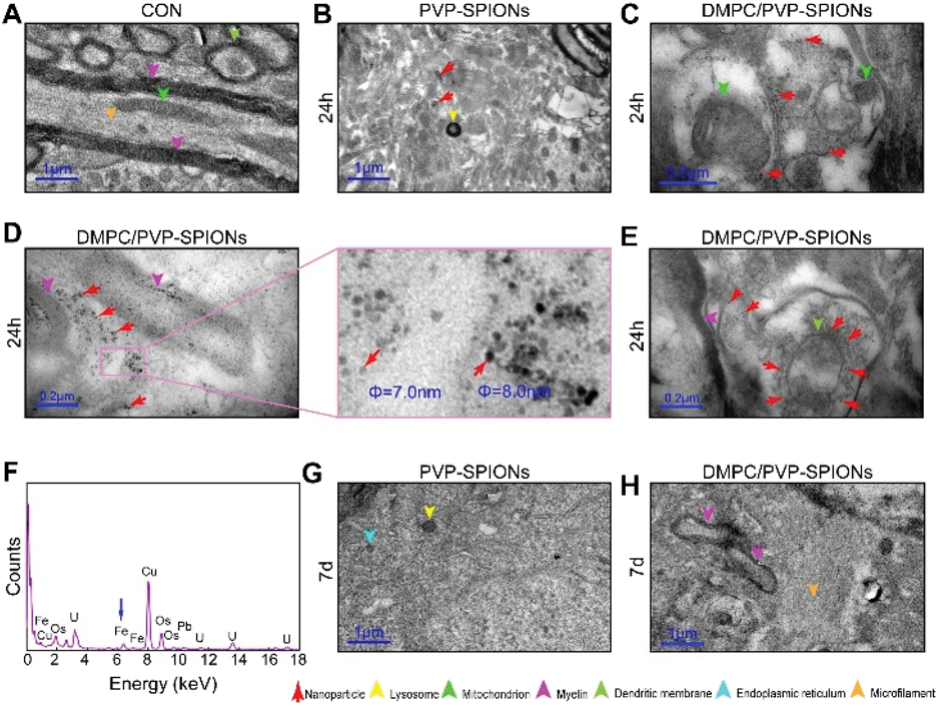
Distribution of nanoparticles in the substantia nigra of CON (A), after 24 h injected with PVP-SPIONs (B), after 24 h injected with DMPC/PVP-SPIONs (C–E), EDS elemental analysis (F), after 7 d injected with PVP-SPIONs (G), after 7 d injected with DMPC/PVP-SPIONs (H).

**Fig. 8:**
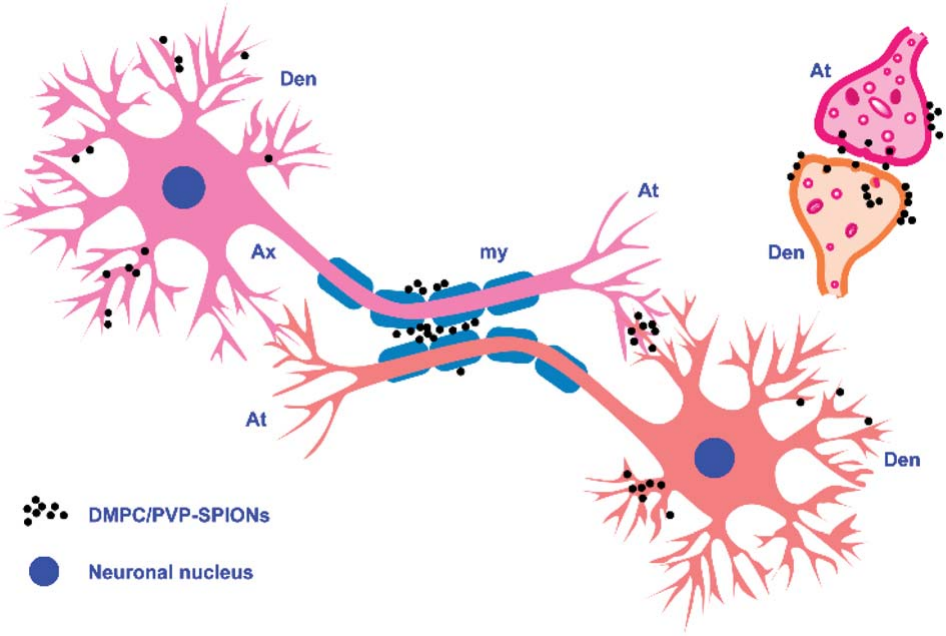
Schematic diagram of the subcellular distribution of DMPC/PVP-SPIONs.

Fig. 7A shows the TEM pictures of the myelin sheath (my), axons (Ax), dendrites (Den), microtubules (m), and mitochondria (mit) in the substantia nigra. PVP-SPIONs were mainly found in the lysosome (Ly) of the cells in the substantia nigra after 24 h, while few were found in other organelles (shown by the red arrows in Fig. 7B). The dense distribution of PVP-SPIONs in the lysosome body can be found in the local enlargement picture (Fig. 7B). On the other hand, DMPC/PVP-SPIONs were mainly distributed on the cellular bio-membranes, such as dendrite (Den) membranes, and near the synaptic cleft between the axonal terminals (At) and dendrites (Fig. 7C–E) show that lots of DMPC/PVP-SPIONs were also found on the outermost membrane layer of the myelin sheath and the area between the two myelin sheaths. The myelin sheath is distributed in regular intervals along the axons of the nervous system;^31,32^ it is a part of neurons along with dendrites, axons, and neuronal bodies. The particle sizes of two particles selected at random by ImageJ were about 7.0 nm and 8.0 nm, which are consistent with the particle sizes of DMPC/PVP-SPIONs. The local magnification picture shows that the DMPC/PVP-SPIONs were homodisperse, and there was no obvious agglomeration of nanoparticles. There were also a few nanoparticles within the dendrite (Fig. 7E), which shows that the nanoparticles have entered the neurons. Fig. 8 is a schematic view of the subcellular distribution of nanoparticles in substantia nigra injected with DMPC/PVP-SPIONs.

The nerve structure in the substantia nigra implanted with DMPC/PVP-SPIONs and PVP-SPIONs was the same as compared with the CON, indicating good biocompatibility of the nanoparticles. The typical X-ray by EDS technology was collected inside the red dotted frame (Fig. 7D). Fig. 7F shows a strong peak of Fe Kα in this region, indicating that the black particles in this region are iron oxide nanoparticles, and peaks of other elements such as Cu, Pb, and U are derived from copper nets and dyes. Fig. 7G and H represent the subcellular structures of the substantia nigra 7 d after the injection of PVP-SPIONs and DMPC/PVP-SPIONs, respectively. However, nanoparticles were not found by TEM in these subcellular structures.

**Fig. 9:**
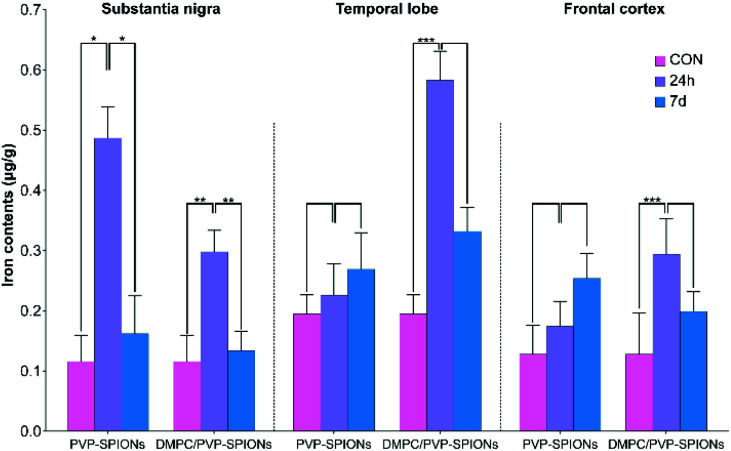
Iron contents in different brain regions (substantia nigra, temporal lobe, and cerebral cortex) at 24 h and 7 d after injection of PVP-SPIONs and DMPC/PVP-SPIONs.

The iron contents of different brain regions (the substantia nigra, temporal lobe, and cerebral cortex) were quantified by ICP-OES (the method can be found in the ESI†) to explore the diffusion of PVP-SPIONs and DMPC/PVP-SPIONs after they were injected into the substantia nigra. Table 2 and Fig. 9 show the effects of the surface modification on the diffusion of iron in rat brains at 24 h and 7 d after the stereotactic injection of SPIONs. The iron content in the substantia nigra after 24 h of injection with DMPC/PVP-SPIONs was less than for PVP-SPIONs. But the iron contents in the temporal lobe and frontal cortex after 24 h of injection with DMPC/PVP-SPIONs were higher than for PVP-SPIONs, which indicates the promoted transport of DMPC/PVP-SPIONs.

**Table 2:** Iron contents (μg Fe per g tissue) in the left brain extracted from three groups of rats injected with PVP-SPIONs and DMPC/PVP-SPIONs after 24 h and 7 d

Group		CON	PVP-SPIONs	DMPC/PVP-SPIONs
Substantia nigra	24 h	115.7 ± 43.0	486.8 ± 52.0	298.0 ± 35.7
	7 d		162.2 ± 62.7	133.8 ± 32.2
Temporal lobe	24 h	194.8 ± 31.8	225.8 ± 51.9	583.1 ± 48.0
	7 d		269.1 ± 60.6	332.1 ± 39.9
Frontal cortex	24 h	128.2 ± 47.7	174.4 ± 41.3	294.0 ± 58.4
	7 d		254.0 ± 41.1	198.9 ± 32.6

The iron contents in the temporal lobe, which is near the substantia nigra, after 24 h of injection with DMPC/PVP-SPIONs was significantly increased and was more than that following injection with PVP-SPIONs. However, the iron content in the cerebral cortex, far away from the substantia nigra, was relatively low, regardless of the time period. The results show that DMPC could promote the diffusion of SPIONs from the substantia nigra to nearby brain regions.

The surface chemistry of nanoparticles plays a crucial role in transportation in the brain, cell distribution, and biocompatibility. The strong interaction between cell membranes and nanoparticles also causes fractures of the lipid bilayer.^33^ It has been reported that aggregation of iron in the brain can easily lead to neurological disorders.^34^ In general, the electrostatic interaction between electronegative cell membranes and electropositive nanoparticles results in the agglomeration of the nanoparticles. In previous research,^35^ DMPC were modified on the surface of positively charged PEI/PEG-SPIONs and implanted into the substantia nigra. After 24 h, it was found that they tended to distribute on the myelin sheath and cell membrane. However, agglomeration and high local concentrations of nanoparticles appeared, and nanoparticles with a positive charge are more likely to induce oxidative stress than those with a negative charge.^36^ Here, DMPC was used to modify PVP-SPIONs. The zeta potential of DMPC/PVP-SPIONs showed a weak negative electrical charge, and no large aggregations were found in the brain. The interaction between non-agglomerated negatively charged nanoparticles and cell membranes has little impact on the fluidity and structure of the membranes.^37^

The results of this work show that nanoparticles in brain tissues were gradually removed and degraded by the brain system over time. Degradation of iron oxide particles is likely to occur in lysosomes because the lysosome chamber is composed of many acidic vesicles (pH = 4–5). Low pH and the existence of reductive compounds such as glutathione, ascorbic acid, and cysteine in lysosomes can promote the degradation of SPIONs and lead to the production of ferrous ions.^38^ Divalent metal transporter 1 (DMT1) exports ferrous ions to the cytoplasm, where they can be used for the synthesis of iron-containing proteins. In addition, SPION-derived ferrous iron catalyzes the formation of reactive oxygen species (ROS).^39^ Excessive iron ions are stored in lysosomal ferritin as a component of lipofuscin; each molecule of ferritin can store about 4500 atoms of iron.^40^ Because cells do not have the capacity to eliminate the lysosomal iron that links to lipofuscin, even if the absorption of iron is effectively regulated, it could slowly accumulate over time.^38^ At the same time, the nanoparticles that are not degraded by lysosomes might escape and spread to other brain regions through the pathways of the cerebral vascular wall, cerebrospinal fluid, or axon pathway.^41^Fig. 7C–E show the distribution of DMPC/PVP-SPIONs in myelinated axons, and they are also found near axonal terminals and within the dendrites, which are related to the transport of nanoparticles in the nervous system.

In summary, injected DMPC-modified nanoparticles were effectively distributed on the bio-membrane after 24 h. The *in vitro* experiments confirmed that DMPC enables SPIONs to easily pass through the cell membrane, indicating that they might have a strong diffusion ability in the myelin sheath due to a similar phospholipid composition. The low-toxicity, non-agglomerated, and fast-transporting nanoparticles introduced in this research have good prospects for application in drug delivery.

## Conclusions

DMPC/PVP-SPIONs were monodispersed in the brain and dispersed in the axon, myelin sheath, and dendritic membrane. DMPC can promote the transport of nanoparticles in the brain and enter cells due to similarities with the structure of the cell membranes. DMPC/PVP-SPIONs with good biocompatibility and biodegradability have potential applications in drug delivery or for activating neuron membrane channels.

## Ethical statement

All animal procedures were performed in accordance with the Guidelines for Care and Use of Laboratory Animals of Guilin Medical University and experiments were approved by the Animal Ethics Committee of Guilin Medical University (no. 2020-0015).

## Conflicts of interest

There are no conflicts to declare.

## Supplementary Material

RA-011-D1RA01737C-s001
