# Supplementary material for: 1,2-Dimyristoyl-sn-glycero-3-phosphocholine promotes the adhesion of nanoparticles to bio-membranes and transport in rat brain
Source: RSC Adv. 2021 Nov 3;11(56):35455–62. doi: 10.1039/d1ra01737c (PMC9043267; doi:10.1039/d1ra01737c)
Supplement: RA-011-D1RA01737C-s001 [file RA-011-D1RA01737C-s001.pdf]

Electronic Supplementary Material (ESI) for RSC Advances.
This journal is © The Royal Society of Chemistry 2021

1, 2-Dimyristoyl-sn-glycero-3-phosphocholine promotes the adhesion of nanoparticles to bio-membrane and transport in rat brain

Supplementary Information

Materials: PEG (Mw=1000) were obtained from Xi-long Science (Shantou, China). PVP (Mw=8000) and DMPC were purchased from Aladdin (Shanghai, China). $Fe(acac)_3$ was from TCI (Shanghai, China). RPMI Medium 1640, Penicillin-Streptomycin, Fetal Bovine Serum (FBS), and Heat-Inactivated Horse Serum (HI-HS) were obtained from Gibco (USA). Cell Counting Kit-8 (CCK-8) was purchased from Dojindo (Japan). 96-well microtiter plates, 6-well cell culture plates, and 15 mL plastic centrifuge tubes were from JET (Guangzhou, China). A 25 cm^2 breathable cell culture bottle was obtained from NUNC (Denmark). Phosphate Buffer Saline (PBS) was from LEAGENE (Beijing, China).

Synthesis of nanoparticles： PEG were heated and dissolved into a transparent liquid at 65 ℃. 15 g of PEG was added into a 50 mL three-mouthed flask, to which 0.3 g PVP was then added. The three-mouthed flask was placed on a heater with a temperature controller (AI-518/518P, Xiamen, China), and the flask was flushed with argon atmosphere. 0.7 g $Fe(acac)_3$ was added to the liquid after the reactants in the three-mouthed flask were heated to 80 ℃ for 10 min. This process requires indoor humidity below 55% to prevent the sample from being oxidized by moisture. After which, the temperature was raised to 260 ℃ for 1 h. After which, the heat source was removed, and the product was allowed to naturally cool down to 55 ℃. The resulting PVP-SPIONs were washed with toluene and acetone successively until the supernatant was colorless and transparent in a clean beaker. The residual toluene and acetone in the PVP-SPIONs were filtered by LS columns (Miltenyi Biotec, Germany), then dispersed in deionized water.

Characterization of SPIONs: Phase characterization of the SPIONs was analyzed by X-ray powder diffraction (XRD, X'Pert PRO, Netherlands). The magnetic properties of the SPIONs were measured by the physical property measurement system (PPMS, PPMS-9, USA). Thermogravimetric analyses (TGA) of the SPIONs were measured with TA Instruments (Q500, USA). Hydrodynamic diameter and zeta potential analysis were determined by nanometer and a zeta potential analyzer (ZEN3600, UK). The subcellular distribution of the SPIONs was observed by Transmission Electron Microscopy (TEM), and iron content in the substantia nigra was determined by inductively coupled plasma optical emission spectrometer (ICP-OES, Optima 7300DV, USA). Surface structure analysis of the SPIONs was tested by Fourier transform infrared spectroscopy (FT-IR, Nexus 470, USA) and X-ray photoelectron spectroscopy (XPS, ESCALAB 250Xi, USA). Cytotoxicity of the SPIONs was detected by an ELISA Microplate Reader (BioTek ELx800, USA).

Cell culture: PC-12 cells after resuscitation were cultured in about 5 mL RPMI 1640 complete medium (including 85% RPMI Medium 1640, 5% FBS, and 10% HI-HS) with 1% Penicillin-streptomycin in a 25 cm^2 breathable cell culture bottle and subsequently placed in a carbon dioxide incubator (311, USA) containing 5% carbon dioxide and 95% oxygen, with a constant temperature of 37 ℃.

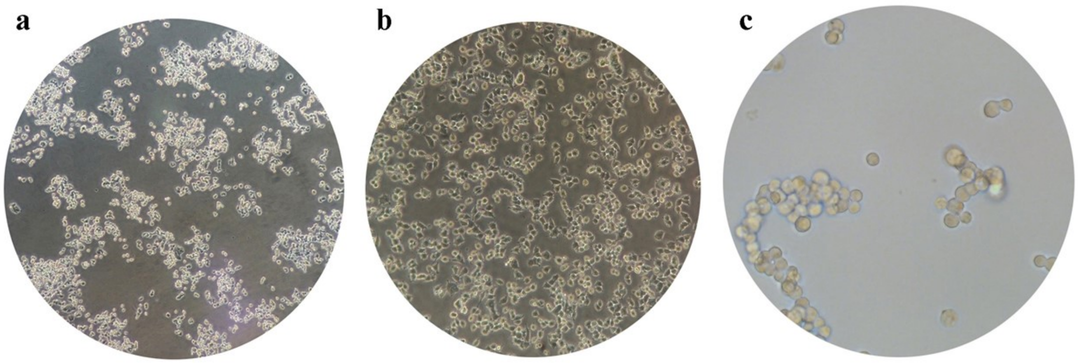

Fig. S1 PC-12 cells immediately after resuscitation (a), after three days of culture (b), and under 20× lens (c)

The procedure for TEM biological sample preparation: The tissue or cell specimens were observed by TEM. For biological TEM analysis, the specimens were fixed in 2.5% glutaraldehyde (Sinopharm Chemical Reagent, China), then specimens were cut into about 1 mm^3 piece and further fixed with 1% osmium tetroxide (SPI Supplies, USA) in 0.1 M PBS buffer for

2 h. The specimens were then dehydrated gradually in 50–100% anhydrous ethanol for 15 min. The specimens were embedded in Epon-Araldite (SPI Supplies, USA) and were heat-treated at 37 ℃/12 h, 45 ℃/12 h, 60 ℃/48 h. The specimens were cut into ultrathin sections with an ultramicrotome (Power Tome-XL, USA). The ultrathin sections were put on copper grids (200 mesh) and dropped with uranyl acetate (SPI Supplies, USA) and lead citrate (SPI Supplies, USA) and then subjected to TEM observation.

Double staining: 1% Uranyl acetate and lead citrate (0.03 g dissolved in 0.1 M NaOH).

(1) Tissue slice staining: First, the copper net with tissue slices was stained with 1% uranyl acetate, using a dyeing time of 7 min. Then, it was rinsed 3–5 times in double evaporated water. After drying, it was stained with lead citrate (this process should minimize the amount of carbon dioxide) and dyed for 2 min, then dried.

(2) Cells slice staining: The same procedure was followed for staining with tissue sections, but the dyeing time was halved.

Determination of iron contents in rat substantia nigra: The substantia nigra, temporal lobe, and cerebral cortex of the injected side of the brain were selected for the ICP-OES measurements of iron contents. First, the tissues from the different groups were dried at 105 ℃ to obtain a constant weight. 1 mL of concentrated nitric acid and 0.2 mL of concentrated hydrochloric acid were added to the weighted sample in a tube and heated at 80 ℃ for 2 h. Then, 0.1 mL of Triton X-100 (Sinopharm Chemical Reagent, China) was added to the tube, and it was kept for another 1 h at 80 ℃ to further destroy the cell membrane. After digestion, the solution was diluted to 10 mL for ICP-OES tests.

FT-IR spectroscopic analysis: Fig.3b shows the FT-IR spectroscopic analysis of the SPIONs, DMPC, and DMPC-SPIONs, and the results further verified DMPC conjugation. A typical Fe-O bond was 580 cm^{-1}, which indicated that the polymer was connected to the SPIONs in the form of a Fe-O bond. The absorption peaks at 2912 cm^{-1} represent C-H groups in PEG, PVP, and DMPC. As shown in Fig.3b,

DMPC and DMPC-SPIONs have absorption peaks at 1732 cm^{-1} and 964 cm^{-1}. Meanwhile, both DMPC and DMPC/PVP-SPIONs have strong absorption peaks at 1239 cm^{-1} and 1070 cm^{-1}, showing the stretching vibration of P-O, and indicating that DMPC has been successfully modified on the SPIONs. The absorption peak at 1385 cm^{-1} represents the symmetrical stretching vibration of COO-. The FT-IR results indicate that DMPC is attached to the surface of the PVP-SPIONs.

XPS spectra analysis: Fig.3c shows the Fe 2p spectrum corresponding to Fe $2p_{3/2}$ (710.3 eV) and Fe $2p_{1/2}$ (724.6 eV) peaks. Fig.4d shows the P 2p peak of elemental phosphorus with a bond energy of 133.3 eV, indicating that elemental phosphorus exists on the P-O bond, which further indicates that DMPC has been successfully modified on the nanoparticles.

Calculation of Modification Quantity: Fig.5a shows that the weight loss rate of the PVP-SPIONs and DMPC/PVP-SPIONs was 35.13% and 58.56%, respectively. Assuming that the mass of the DMPC in a DMPC/PVP-SPIONs is A, the mass of PEG/PVP in a PVP-SPIONs is B, and the mass of DMPC/PVP-SPIONs is C, the weight loss rate of DMPC/PVP-SPIONs is W_1 = 58.56% and the weight loss rate of PVP-SPIONs is W_2 = 35.13% after the removal of water by thermogravimetric analysis. The formula $A/C = (W_1-W_2)/(1-W_2)$ can be obtained from $(A+B)/C=W_1$, $B/(C- A) = W_2$, then the weight ratio of DMPC to DMPC-SPIONs is 36.12% by calculation.
